# Protocol for a systematic review: Universal school‐based programmes for improving social and emotional outcomes in children aged 3–11 years: a systematic review and meta‐analysis

**DOI:** 10.1002/CL2.210

**Published:** 2018-05-10

**Authors:** Paul Connolly, Sarah Miller, Jennifer Hanratty, Jennifer Roberts, Seaneen Sloan

## BACKGROUND

### The problem, condition or issue

Social and emotional competence is vital for forming and sustaining good relationships, solving everyday problems, adapting behaviour and making healthy choices throughout life. It includes self‐awareness, understanding and working with others, controlling emotions and caring about oneself and others. Through social and emotional learning, children and adults are able to develop the skills necessary to work and live effectively in society, yet a significant proportion of pupils struggle with one or more facets of social and emotional development (Doll, Brehm, & Zucker, 2014; Jones & Bouffard, 2012; Zins, 2004). Estimates suggest that between 9.5 and 14.2 percent of children under five experience some form of social and emotional problem which negatively impact their functioning, development and school‐readiness (Brauner & Stephens, 2006).

Underdeveloped social and emotional competencies can have consequences which are far reaching and long lasting (Daly, Delaney, Egan, & Baumeister, 2015; Durlak, 2015; Moffitt et al., 2011). Deficits in basic skills ‐ such as the ability to identify emotions ‐ can affect all stages of the lifespan, including: being rejected by others; exclusion from peer activities; being victimised; and lower peer‐rated popularity (Lemerise & Arsenio, 2000; Leppänen & Hietanen, 2001; Mostow, Izard, Fine, & Trentacosta, 2002). Chronic physical aggression during primary school increases the risk of violence and delinquency through adolescence in boys (Broidy et al., 2003; Nagin & Tremblay, 1999), which in the long term can lead to destructive forms of emotion management, such as alcohol abuse. Poor self‐regulation in childhood is associated with negative outcomes in adulthood including poorer health, social and economic outcomes (Compas, Connor‐Smith, & Jaser, 2004; Daly et al., 2015; Kubzansky, Park, Peterson, Vokonas, & Sparrow, 2011; Moffitt et al., 2011), lack of (Nota, Soresi, & Zimmerman, 2004) and increased criminal behaviour (Henry, Caspi, Moffitt, Harrington, & Silva, 1999).

Social and emotional learning (SEL) programmes aim to intervene early to address skill deficits and equip children with the social and emotional competencies they need for life (Eisenberg, Spinrad, & Eggum, 2010; Moffitt et al., 2011). Attention has increasingly turned to facilitating social and emotional learning in the classroom as a means to help children develop skills such as; empathy, emotional regulation and behaviour management strategies, that will enable them to become functioning members of society in adulthood (Humphrey, 2013; Humphrey, Lendrum, & Wigelsworth, 2010).

### What is social and emotional learning?

Social and emotional learning (SEL) has been defined in different ways. Definitions tend to encompass a range of competencies, concepts and areas for development. For example, the American Collaborative for Academic, Social and Emotional Learning (CASEL, 2015) have described and defined five core competencies: self‐awareness; self‐management; social awareness; relationship skills; and responsible decision making. Waters & Sroufe (1983) also describe these social and emotional competencies as being important in enabling children “to generate and coordinate flexible, adaptive responses to demands and to generate and capitalize on opportunities in the environment” (p80). In the UK, The Young Foundation (McNeil, Reeder, & Rich, 2012) have identified a core set of evidence‐based social and emotional capabilities shown to be important throughout the lifespan, including: communication; confidence and agency; planning and problem solving; relationships and leadership; creativity; resilience and determination; and managing feelings. Whilst core competencies vary in their definitions and scope between authors and agencies, a consistent pattern emerges demonstrating the array and reach of social emotional learning and its related competencies.

### The importance of social and emotional learning

It has been evidenced both nationally and internationally (Barry, Clarke, Jenkins, & Patel, 2013; Weare & Nind, 2011; Yoshikawa et al., 2015) that improving social and emotional learning allows children to connect with others and to begin to learn in a more effective way, thereby increasing their chances of success both in school and in life. Many countries have endeavoured to embed SEL into school ethos and culture. For example; in England personal and social development is addressed at the policy and practice level through the inclusion of building character and resilience as a priority by English Department of Education's (*Department of Education*, 2016); In America, CASEL work to support the integration of SEL in education through research, practice and policy; In Australia, SEL has been embedded in the national curriculum under personal and social capability (Collie, Martin, & Frydenberg, 2017).

There is a growing consensus in academic and policy circles regarding the importance of children's social and emotional development and its links to behavioural and health outcomes (Ciarrochi, Deane, & Anderson, 2002; NICE, 2008; Petrides, Frederickson, & Furnham, 2004). Children who fail to achieve developmental milestones associated with SEL may be at risk of failing to make meaningful relationships with their peers and with the school situation (Zins, 2004; Zins & Elias, 2007). Social and emotional outcomes are related to educational outcomes (Duckworth & Seligman, 2005), emotional wellbeing (Eisenberg et al., 2010) and general life trajectories (Daly et al., 2015; Moffitt et al., 2011). Educationally, a range of social and emotional factors have been found to have an impact on educational achievement (Banerjee, Weare, & Farr, 2014; Durlak, Weissberg, Dymnicki, Taylor, & Schellinger, 2011), including willingness to learn, openness to new experiences and one's ability to interact with peers and teachers in the school situation.

Given the increasing emphasis on SEL in both the educational research and policy arenas, there has been increased importance placed on SEL in schools and the need for facilitating optimal development of SE competence in every child. A variety of school‐based programmes have been developed which aim to target, remediate and/or improve social and emotional skills in children, from the very beginning of their educational careers. Within the context of education and schooling, these programmes are particularly important for a number of reasons. Firstly, children do not learn in isolation, but rather construct meaning based on their life experiences (Vygotsky, 1980) and the relationships they have built can either facilitate or impede the learning process (Zachary, 2011; Zeidner, Matthews, & Roberts, 2012). Secondly, social and emotional skills are necessary antecedents for learning and constructing knowledge and can help ensure that children are ready, willing and able to learn. Thirdly, strong social and emotional skills enable children to work together, work with others and work alone in the school setting.

### Moderating factors

There is evidence to suggest that certain groups of children are more likely to have difficulties in specific areas of social and emotional development. Children from socially disadvantaged communities and children who suffer maltreatment, are found to be less likely to develop social and emotional skills in line with their peers (Blair & Raver, 2012). Research on similarities and differences between social and emotional development in boys and girls is equivocal with some studies suggesting girls and boys develop in different ways (Lehmann, Denissen, Allemand, & Penke, 2013) while others conclude that these differences may be exaggerated (Else‐Quest, Higgins, Allison, & Morton, 2012). Finally, age is likely to be a moderating factor. While early intervention has been shown to produce greater impacts this is not the case for all relevant outcomes (Sklad, Diekstra, Ritter, Ben, & Gravesteijn, 2012). Understanding the developmental trajectory of social and emotional skills and identifying key developmental stages may help identify the age at which interventions are likely to confer most benefit.

### Description of the intervention

This systematic review will focus on curriculum based SEL interventions delivered in preschool or primary/elementary schools aimed at improving social and emotional skills among pupils. It will include any universal programme, delivered on a whole‐class or school basis. The interventions' primary goal must be to improve social and emotional competence and be delivered in a pre‐school/kindergarten or primary/elementary school setting as part of the normal school day.

The intervention must be structured, include a taught component and be delivered directly to children and involve the active participation of the child. The aims of the programme must be explicitly related to child gains, as opposed to those which focus solely on teacher competencies or school ethos. The intervention must also be a curriculum based social emotional learning program. Interventions may be delivered by the class teacher, other school personnel or non‐school personnel. Interventions must be delivered for a minimum of one school term.

### How the intervention might work

Whilst interventions are varied in terms of their scope and their delivery, they typically adopt either a preventative or remediative approach and may use a range of theoretical approaches, such as Bronfenbrenner's ecological theory or the achievement model of emotional literacy (Rivers & Brackett, 2010). Many SEL interventions in preschool and primary/elementary school are based upon an implicit or explicit understanding of how social and emotional skills develop among children at this age. Rarely, however, do interventions outline a logic model which clearly sets out how the intervention creates or facilitates change in the desired outcomes. To this end, most interventions encourage and help children recognise and identify emotions or undesirable behaviours. Children are then taught, usually through scene setting, discussion and/or modelling techniques, skills to help them reflect on the problem and think through (and thus modify) the various and alternative actions they might take when faced with a difficult or conflict‐related situation.

A range of programmes have emerged that target children who are not meeting the developmental milestones associated with social and emotional learning. Some of these milestones include, for example: being able to understand complex and simultaneous feelings; consider multiple perspectives; empathise or sympathise with others; and recognise, regulate and express emotions effectively in order to create and sustain personal and social relationships. In so doing, SEL interventions aim to support children to enhance their positive emotions and behaviours, whilst also moderating their negative emotions and associated behaviours.

Programmes such as *PATHS* (Promoting Alternative Thinking Strategies) are implemented widely in a number of countries and are designed to facilitate the development of self‐control, emotional awareness and interpersonal problem‐solving skills. PATHS does this by teaching children strategies to help them regulate their emotional response to a difficult situation by: stopping to reflect on the problem, thinking through alternative solutions and based on this, choosing an appropriate course of action. Here, the programme's aims and objectives are closely tied with the developmental achievements which have been set out in previous literature. Similarly, *Roots of Empathy* – a classroom based SEL intervention ‐ seeks to promote prosocial behaviour and reduce aggressive behaviour by enhancing children's emotional recognition, empathy and emotional regulation. It is a structured curriculum‐based programme that involves a mother and baby coming to class. Children observe the baby's development over the period of a school year and are taught to ‘recognise’ the baby's feelings and reflect on their own and others feelings. Learning about the baby's development, how the baby might be feeling, why they might be upset or happy and observing the loving parent‐child relationship provides children with a model of responsible parenting from which to learn. The intervention relies on a mixture of modelling, discussion and storytelling to teach children about their own and others' emotions and behaviours.

Based on the logic model suggested by CASEL, explicit instruction in SEL skills (provided through the intervention) combined with teacher instructional practices that are both integrated with the academic curriculum and school ethos, should lead to the acquisition of SEL skills, improved attitudes towards self, others and learning as well as an enhanced learning environment. In turn, these changes result in positive social behaviour, fewer conduct problems, lower emotional distress and improved academic performance.

For the purpose of the current review, the co‐primary outcomes will be behavioural, namely: improved prosocial behaviour and fewer conduct problems. Secondary outcomes will include: SEL skills (including emotional regulation, emotional recognition, empathy, problem solving); attitudes (including self‐esteem, peer relationships, enjoyment of school); emotional distress and academic performance.

Whilst certain background or demographic characteristics may moderate the impact of a programme on certain groups of children or families (previously discussed), other programme related factors – such as quality and fidelity of implementation – can also moderate effects. A number of programme factors emerge which could be seen to be important when implementing a programme. As mentioned in Clarke et al's (2015) recent review, parental involvement may be an area which facilitates success of an intervention. Here it was found that when parents were involved and knowledgeable about the process being undertaken children were more likely to make significant gains. Additional factors which may lead to greater successes include teachers and other school staff implementing the programme compared to instructors from outside the school (Durlak et al., 2011, p.13) which can allow children to make greater gains, perhaps because they feel calm and confident with the instructor. There is also some evidence to suggest that structured programmes which incorporate training for those delivering the program, are interactive in nature and which guide young people towards a specific set of goals are more likely to be successful (Smith, Schneider, Smith, & Ananiadou, 2004). Moreover, there is some evidence to suggest that multi‐component programmes may have added benefits compared with single component programs (Adi, Killoran, Schrader McMillan, & Stewart‐Brown, 2007; Catalano, Berglund, Ryan, Lonczak, & Hawkins, 2004; Humphrey et al., 2010; Wells, Barlow, & Stewart‐Brown, 2003). Factors regarding programme design and implementation are also influential, with programmes which were identified as well‐designed and well implemented being the most impactful (Durlak et al., 2011). Stage of programme development may affect the success of the intervention, with efficacy trials conducted under controlled conditions and often led by the programme developer resulting in greater effects than effectiveness trials conducted under more ‘real world’ conditions (Wigelsworth et al., 2016).

These programme content and implementation factors will be documented in the coding framework and, if possible, analysed as moderators of intervention effect.

### Why it is important to do the review

The importance of SEL is now well recognised, and there has been significant growth in the number and type of SEL programmes offered in schools. Existing related reviews either do not focus on broad‐based SEL programs or are currently out of date, or have included studies which used non‐robust methodologies.

The Campbell and Cochrane systematic review libraries were searched in December 2017 for completed and ongoing reviews relevant to this area. This search found a number of relevant ongoing or completed reviews:

- A review of universal school‐based social information processing interventions for aggressive behaviour (Wilson & Lipsey, 2007). This review only included studies published until 2003 and focussed mainly on violence reduction and prevention as opposed to broader social emotional learning
- A review of school‐based programmes to reduce bullying and victimisation (Farrington & Ttofi, 2009). This review only considered evaluations which measured bullying or aggression towards peers but did not consider wider social and emotional skills.
- A review of self‐control programmes for reducing delinquency and problem behaviours (Piquero, Jennings, Farrington, & Jennings, 2010). This review did not have an explicit focus on universal, school‐based programmes (79% of included studies were school‐based, and only a third were universal).
- A protocol for a review of school‐based interventions for reducing disciplinary school exclusion (Valdebenito, Eisner, Farrington, Ttofi, & Sutherland, 2015). This review focuses on school exclusions and thus does not include a wider range of social emotional learning measures.
- A protocol for a review of school‐based executive functioning interventions (Steenbergen‐Hu, Olszewski‐Kubilius, & Calvert, 2017). This review focusses solely on core components of executive function, and aims to use direct executive function outcomes and so does not focus on wider social and emotional learning.
- A review of mindfulness‐based interventions for improving academic achievement, behaviour and socio‐emotional functioning of primary and secondary students (Maynard, Solis, Miller, & Brendel, 2017). This review restricts its focus to interventions making use of mindfulness tools rather than including interventions that use a wider range of techniques to improve social and emotional learning.
- A protocol for a review of practices and programme components for enhancing prosocial behaviour in children and youth (Spivak, Lipsey, Farran, & Polanin, 2015). This review focusses on pro‐social behaviour in the classroom setting, and does not have a specific focus on interventions aimed at the broader range of social and emotional outcomes.
- A review of effective programmes for social and emotional learning (Cocoran & Slavin, title registered January 2016). This review is mainly focused on academic attainment, and how social and emotional programmes impact attainment. This review does not have a focus on social and emotional outcomes.
- A review of the Tools of Mind curriculum for improving self‐regulation in young children (Baron, Evangelou, Malmberg, & Melendez‐Torres, 2017) focused on just one intervention.

In the wider research literature, several other reviews have been conducted in the area of social and emotional learning (SEL) programmes (Browne, Gafni, Roberts, Byrne, & Majumdar, 2004; Clarke, Hussein, Morreale, Field, & Barry, 2015; Durlak et al., 2011; Payton et al., 2008; Sklad et al., 2012; Taylor, Oberle, Durlak, & Weissberg, 2017; Wigelsworth et al., 2016; Wilson & Lipsey, 2007). The most relevant of these is Durlak et al.'s (2011) meta‐analysis, which focused on school‐based programmes and their impact on a number of pupil outcomes including: social and emotional skills and attitudes; positive social behaviour; conduct problems; emotional distress; and academic performance. Durlak et al.'s searches were conducted up until the end of 2007 and thus the findings are now dated. Whilst more recent, Clarke et al.'s (2015) review focussed only on literature published in the UK, did not conduct meta‐analyses and studies were included which did not include control groups. More recently, (Wigelsworth et al., 2016) examined the impact of developer involvement, trial stage and transferability on universal SEL interventions. This review represents an important step in explicating the complex relationships between efficacy and effectiveness, programme fidelity and transferability. However, it was limited to studies reported in English between 1995–2013, excludes pre‐school programmes and excluded studies not reporting means and standard deviations, which misses an opportunity to identify gaps and bias in reporting of conducted trials. Finally, Taylor and colleagues (Taylor et al., 2017) conducted a meta‐analysis of follow‐up effects of SEL programs including studies reported in English up to December 2014. This review provides an analysis of the longer term impacts of SEL programs but does not include recent studies or studies in a language other than English.

Since these reviews have been published, the review team is aware of a number of new evaluations conducted on interventions aimed at pupil emotional wellbeing and behaviour through universal social and emotional learning programmes. An up‐to‐date systematic review including more recently conducted studies is therefore needed. Additionally, whilst there are a number of ongoing or completed narrative reviews which focus on individual programmes, specific social and emotional outcomes or specific niches or facets of socio‐emotional development, the reviews which have been (or are being) completed currently tend not to systematically review, map and assess the wide range of social and emotional learning programmes currently being used in schools for children aged 3–11. This proposed review will therefore be more wide‐ranging and inclusive and will allow for the inclusion of more recent literature emerging in the field.

Finally, this review is seeking to address a broader set of questions regarding the overall impact of universal school‐based SEL programmes and, within this, has a particular focus on comparing the effectiveness of different types of intervention, and for different subgroups, in order to determine whether there are any underpinning programme‐specific components that are associated with greater effects. These objectives require a broad‐based review that has not been attempted by any of the reviews listed above.

## OBJECTIVES

The objectives of the review are:

1) To identify, appraise and synthesise evidence from randomized and cluster randomized trials on the effectiveness of universal, school‐ based social and emotional learning programmes for young children age 3–11 as compared to no intervention or inactive control, for improving children's social and emotional skills and behavior.
2) To identify and classify the core components of programmes and to assess which of these components, if any, can be considered as ‘active ingredients’ in terms of their presence or absence being associated with differential programme effectiveness.
3) To ascertain whether fidelity of delivery and the stage of the programme's development are associated with programme effectiveness.  
4) To assess if the programmes are differentially effective for different subgroups in relation to age, gender and socio‐economic background.
5) To develop a set of clear and specific recommendations, based on the evidence reviewed, to guide the further development and delivery of universal school‐based social and emotional learning programmes.  

## METHODOLOGY

### Characteristics of the relevant studies

Given the broad definition of social and emotional learning and the wide variety of school‐based interventions that now exist, studies will be varied in terms of their design, methodology, implementation, outcomes and measurement. Four representative studies that could potentially meet the inclusion criteria for the current review, are detailed below.

1. Domitrovich, Cortes and Greenberg (2007) report a cluster randomised controlled trial evaluation of the PATHS (Promoting Alternative THinking Strategies) curriculum (Kusché & Greenberg, 1994). This classroom‐based curriculum is designed to reduce problem behaviour whilst promoting social competence. This wait list control trial involved twenty classrooms in Pennsylvania, ten randomly allocated to each of the control and intervention groups. The intervention was delivered to children of nursery age (between three and four) by a trained class teacher, who implemented weekly lessons (30) and extension activities across a nine month period
2. Snyder, Vuchinich, Acock, Washburn and Flay (2012) conducted a randomised controlled trial on Positive Action, a character development programme which aims to improve school quality, improve academics, student behaviour, and character and to mitigate problem behaviours. This universal, school wide programme includes a school‐wide climate development component, teacher/staff training, a coordinator's manual, school counsellor's programme, and PA coordinator/committee guide; and family‐ and community‐involvement programmes. The sequenced elementary curriculum consists of six units which are divided into 140, 15‐ to 20‐minute lessons per grade, per academic year, provided by classroom teachers. This trial involved 20 racially/ethnically diverse schools and was conducted between 2002 and 2006. Teacher, parent and student data were collected in pre and post tests and additional, school‐level archival data were used to examine programme effects at one year post‐trial.
3. Mendelson et al., (2010) conducted a randomised trial assessing the feasibility, acceptability, and preliminary outcomes of a pilot school‐based mindfulness and yoga intervention. An existing intervention was manualised and structured into sessions which ran for four days a week for twelve weeks. Two schools took part, with half of the children in each school being assigned to the intervention group, which consisted of around 50 children.
4. Holen and colleagues (Holen, Waaktaar, Lervåg, & Ystgaard, 2012) evaluated Zippy's Friends, a universal school based programme that aims at strengthening children's coping skills, with 1,483 children in 35 schools aged between seven and eight. This manualised, structured programme is delivered in 24 weekly lessons and is based on six stories about three cartoon characters. The main objective of the programme is to prevent psychological problems by increasing children's coping repertoire and giving them various ways of coping with problems.

### Criteria for including and excluding studies

#### Types of study designs

Randomised controlled trials or cluster randomised controlled trials will be included. We anticipate a large number of included studies and so can reasonably restrict our analysis to only the highest quality research designs.

#### Types of participants

Children attending preschool or primary/elementary schools. This population is typically between the ages of three and eleven years.

Where a study includes participants from both preschool or primary schools and secondary schools reasonable attempts will be made to extract or source the data for only the children attending preschool/primary/elementary school from published and unpublished documentation. If data us unavailable authors will be contacted to request summary statistics for only the participants of interest.

Studies which are located in special schools or which focus on children with identified special educational needs or social, emotional or behavioural difficulties will be excluded.

#### Types of interventions

Studies will be included if the intervention is a universal, school‐ or classroom‐based programme which is delivered, via a set curriculum, to a whole class or whole school and which primarily aims to improve social and emotional competencies of children. The intervention must be a curriculum‐based social emotional learning programme.

The intervention must be delivered directly to children and involve the active participation of the child. The aims of the programme must be explicitly related to child gains, as opposed to those which focus solely on teacher competencies or school ethos.

Interventions may be delivered by the class teacher, other school personnel or non‐school personnel. Interventions must be delivered for a minimum of one school term.

Studies must include an inactive comparison condition that could include;

- **No treatment**.
- **Treatment as usual** where pupils receive their normal level of support or intervention. Details of what this consists of will be extracted.
- **Waiting list** where schools or classrooms are randomly assigned to receive the intervention at a later date. Details of what happens to waitlisted participants will be extracted.
- **Attention control**, where participants receive some contact from researchers but both participants and researchers are aware that this is not an active intervention.
- **Placebo** where participants perceive that they are receiving an active intervention but the researchers regard the treatment as inactive.

Studies with an inactive control compared to two or more intervention arms can be included and the sample size of the control group will be divided by the number of eligible intervention arms to avoid double counting control group participants in any meta‐analysis.

The following types of studies will be excluded:

- Studies in which the SEL programme was delivered in an after‐school setting or without a school‐based component (e.g. in youth clubs, summer clubs, sports or social clubs, or through parenting groups);
- Studies in which the SEL programme targets specific groups associated with the outcomes of interest (e.g. pupils with special educational needs); and
- Studies in which the programme is not delivered directly to children (e.g. interventions aimed at changing the behaviour or attitudes in teachers without any direct involvement of children).
- Studies in which the programme is not delivered according to a pre‐specified curriculum or manual (e.g. school providing leisure facilities to pupils).
- Studies in which the programme is delivered as a one off or infrequent intervention.

Kratochwill et al. (Kratochwill, McDonald, Levin, Scalia, & Coover, 2009) represents an example of a study that is likely to be excluded. This study evaluated the Families and School Together (FAST) programme (McDonald et al., 1997). Here, children were recruited from schools and the programme delivered in the school setting. The programme aimed to improve a number of outcomes, including parental involvement, child behaviour and teacher perceptions of attainment. To do this it delivered eight weekly sessions where children and parents took part in games and activities together. Whilst this study does include some aspects of social and emotional development, the main focus was on parents who are working alongside their children. This is a multi‐family, group intervention, rather than one which focusses on the child and their social and emotional development. As there is no direct taught element which is delivered to the child this study would not meet the inclusion criteria

#### Types of outcome measures

**Primary outcomes**

1. Prosocial – behaviour.
2. Conduct problems.
**Secondary outcomes**
3. SEL skills including emotional regulation, emotional recognition, empathy, problem solving.
4. Attitudes towards self and others including self‐esteem, peer relationships, enjoyment of school.
5. Emotional distress, such as anxiety, depression social withdrawal.
6. Educational attainment.
7. Adverse effects.

In SEL trials it is rare that adverse effects are considered and so we will not select adverse outcomes *a priori*. Instead we intend to extract any data on adverse outcomes and to provide a narrative summary of potential adverse effects.

Outcome measures vary widely in terms of quality and validity. We will exclude treatment inherent measures as they risk overestimation of effect size (Slavin & Madden, 2008). The minimum standard will be that for any instruments used in included studies a full description of the scale and its scoring as well as associated reliability statistics are available.

### Search strategy

In order to find all eligible studies the following data sources will be searched and consulted. This comprehensive search will include multiple electronic databases, research registers, grey literature sources, and reference lists of reviews and relevant studies. We will not restrict the study selection in terms of language, date or publication status. Each of the following databases and trial registries will be searched using the search strings set out in appendix 1:

a) British Education Index
b) Education Abstracts (EBSCO)
c) ERIC
d) MEDLINE
e) PsycINFO
f) Web of Science/ Knowledge Database Science Citation Index
g) Web of Science/ Knowledge Database Social Science Citation Index
h) ClinicalTrials.gov
i) National Registry of Evidence‐based Programs and Practices

Relevant reviews will be searched for in the following databases;

j) Database of Abstracts of Reviews of Effectiveness
k) The Campbell Library
l) Cochrane Collaboration Library
m) Evidence for Policy Practice Information and Coordinating Centre (EPPI‐Centre)

We will also search grey literature and the following databases and websites will be used:

n) CASEL
o) Education Endowment Foundation
p) Google Scholar – using a series of searches and screening the first 2 pages of results for each search
q) ProQuest Dissertations and Theses
r) WorldCAT
s) OECD Education Library
t) Opengrey
u) World Health Organization

Reference lists of relevant reviews and included studies will be screened and forward citation searching of included studies will be carried out through Google Scholar. These web searches will be limited to the first 2 pages of search results. Prominent authors in the field will also be contacted. In addition, a final step towards the end of analysis, a manual search of the most recent issue(s) of key journals will be conducted. To do this, the top 10 journals that have provided included studies so far, will be identified and their most recent issues will be checked.

One reviewer will conduct the database searches, remove duplicates and obviously irrelevant records. We anticipate that the searches will result in a very large number of records to screen and so to ensure robustness, each report will be screened by title and abstract by two reviewers. Potentially eligible studies will then be retrieved in full text form and two reviewers will screen each full text. Any disagreements will be discussed with the wider review team until a consensus is reached.

### Search terms and keywords

The search strategy has been developed using a modified version of the pearl harvesting approach. First, one author (JR) extracted keywords from ten randomly selected relevant studies. Each of the terms extracted were then searched individually within the thesauri in ERIC, British Education Index and Psycinfo and all additional terms added to the list of possible search terms relating to either: the participants; intervention setting; study design; or outcomes of interest. All authors then suggested additional terms that had not been identified (e.g. nursery school did not appear in any article of thesauri). Two authors (JR and JH) then screened the full list of terms and removed any duplicate, overlapping or irrelevant terms. The search string samples below therefore were generated by a combination of terms originating from the literature, terms originating from the review team and terms originating from a thesauri search.

A combination of four search strings will be used relating to: 1) participants; 2) the intervention setting; 3) the study design; and 4) the outcomes of interest. A sample search strategy is provided in appendix 1. Search strings and search limits will be modified to be suitable for each database. Search for exact phrases or proximity searching will be used to increase search specificity. In addition searches for a number of known named interventions will also be undertaken (see appendix 1).

### Description of methods used in primary research

All included studies will be randomised or cluster‐randomised controlled trials.

### Criteria for determination of independent findings

It is important to ensure that the effects of an individual intervention are only counted once and the following conventions will therefore apply.

Where there are **multiple measures reported for the same outcome**, this will be dealt with by calculating an average effect size within each study for each outcome. A simple average effect size will be calculated by first calculating the effect size for each measure of a given outcome and then averaging these effect sizes within each study. The exception will be any treatment inherent measures of the outcome of interest, these measurements will be discarded as they risk overestimating the treatment effect.

Where the **same outcome construct is measured but across multiple time domains**, such as through the collection of both post‐test and further follow‐up data, the main analysis will focus on synthesising the evidence relating to effect sizes at immediate post‐test. Any subsequent measures of outcomes beyond immediate post‐test will be meta‐analysed and reported separately.

Studies comparing **multiple treatment and control arms** will be discussed with the full author team to decide if eligible intervention arms are similar enough to combine and compare as if they are one intervention group. If not, each intervention arm will contribute separate effect sizes to the meta‐analysis and the control group sample size will be split by the number of intervention arms included to avoid double counting of control participants.

In the case of **multiple cohorts** appearing in one study we will calculate a simple average, as described above, for the omnibus meta‐analysis. If different cohorts in a study fall into different subgroups then they will be considered separately in subgroup analysis but no overall summary of effect will be calculated combining subgroups in those cases. If there are sufficient eligible studies reporting multiple and dependent effect sizes (i.e. occurring in more than 20 eligible studies) then robust variance estimation will be employed. This technique calculates the variance between effect sizes to give the variable of interest a quantifiable standard error. It has been shown to calculate correct results with a minimum of 20–30 individual studies (Hedges, Tipton, & Johnson, 2010) although it performs better with an increased quantity of studies.

### Details of study coding categories

Once eligible studies have been found, an initial analysis of programme descriptions will be undertaken for all eligible programmes. This will be used to identify the core components of programmes and to develop an overarching typology and coding frame for these. From previous studies, and as referenced in the earlier discussions of existing literature, it is anticipated that such components are likely to include:

- Duration and intensity of the programme.
- Whether the programme was delivered by external facilitators or internally by teachers or other school staff.
- Whether the programme included training and/or ongoing support for the facilitators.
- Whether the programme explicitly included parental involvement.
- How well structured the programme is.
- Whether the programme has a specific set of goals that it guides participants towards.

Alongside extracting data on programme components, descriptive information for each of the studies will be extracted and coded to allow for sensitivity and subgroup analysis. This will include information regarding:

- the study characteristics in relation to: design, sample sizes, measures and attrition rates, who funded the study, and whether the study was conducted by a research team associated with the programme or an independent team.
- the stage of programme development, for example whether it is a new programme being piloted or an established programme being replicated.
- the level to which the programme was delivered with fidelity using information provided in the study report to categorise each programme as being delivered as with ‘high’, ‘medium’ or ‘low’ fidelity.
- the participants' characteristics in relation to age, gender and socio‐economic background.

A coding framework has been devised and piloted (see appendix 2) and will be refined as necessary based on the above. Coding will be carried out by trained researchers. Each study will be coded by two members of the review team independently and discrepancies will be discussed and a consensus agreed.

Quantitative data will be extracted to allow for calculation of effect sizes (such as mean change scores and standard error or pre and post means and standard deviations). Data will be extracted for the intervention and control group on the relevant outcomes measured in order to assess the intervention effects. If study reports do not contain sufficient data to allow calculation of effect size estimates authors will be contacted to obtain necessary summary data, such as means and standard deviations or standard errors.

Assessment of methodological quality and potential for bias will be conducted using The Cochrane Risk of Bias tool. This is a standard tool, which takes the forms of a series of questions about the randomisation procedures and blinding. The overall quality of evidence relating to the primary outcomes will also be summarised using the GRADE system (Ryan & Hill, 2016).

### Statistical procedures and conventions

#### Calculation of effect sizes

It is anticipated that most outcomes reported will be based upon continuous variables and so the main effect size metric to be used for the purposes of the meta‐analyses will be the standardized mean difference, with its 95% confidence interval. Within this, Hedges' g will be used to correct for any small sample bias. Where other effect sizes have been reported, such as Cohen's d or risk ratios (for dichotomous outcomes) these will be converted to Hedges' g for the purposes of the meta‐analysis using formulae provided in the Cochrane Handbook (Higgins & Green, 2011).

As most studies are likely to involve group‐level allocation, where possible, data will be included which have been adjusted to account for the effects of clustering, typically through the use of multilevel modelling or adjusting estimates using the intra‐cluster correlation coefficient (ICC). Where the effects of clustering have not been taken into account, estimates of effect size will be adjusted following guidance in the Cochrane Handbook. If ICC is not reported external estimates will be obtained from studies that provide the best match on outcome measures and types of clusters from existing databases of ICCs (Ukoumunne, Gulliford, Chinn, Sterne, & Burney, 1999) or other similar studies within the review.

#### Approach to meta‐analysis

Given the diverse range of interventions that this review is likely to find, random effects models, using inverse‐variance estimation, will be used as the basis for meta‐analysis. The analysis will be conducted using Stata 14 and the range of commands externally developed to conduct meta‐analysis with Stata such as metan and metareg (Palmer & Sterne, 2016). Heterogeneity will be measured and reported through the Q, I2 and Tau2 statistics.

#### Main effects and sensitivity analyses (Objective 1)

The main effects analysis, synthesising the evidence in relation to the effects of SEL programmes in general, will be undertaken using the approach to meta‐analysis outlined above for each primary and secondary outcome in turn.

For each outcome, the following sensitivity analyses will also be undertaken to assess whether there are potential influences relating to:

1. Studies that appear to exert an undue influence on findings.
2. Whether the study was undertaken by any of the review authors.
3. Levels of attrition.
4. Study quality (Studies with a “high” or “unclear” risk of bias on 3 or more of the 7 risk of bias domains in the Cochrane Risk of Bias assessment will be coded as low quality).
5. Level of involvement of the program developer in the evaluation (led by the programme developer, developer involved but not leading, independent).
6. Who funded the study (i.e. was the study funded by the programme developers or an independent funder).
7. Stage of the programme's development (efficacy, effectiveness).

In relation to studies that appear to exert an undue influence, a further meta‐analysis will be conducted for each outcome that omits these studies to assess whether their inclusion exerts an influence on the findings.

For the remaining six potentially influencing factors, a meta‐regression will be conducted for each outcome. In each case, meta‐regression will only be used if there are the minimum prerequisite of ten studies (Borenstein, Hedges, Higgins, & Rothstein, 2009). The independent variables added to the regression model in each case will consist of dummy variables associated with the potential influences. The statistical significance of the coefficients in the resultant models for each of the dummy variables will be used to assess, formally, whether there is a notable influence on the findings with regarding to the above factors. Overall conclusions regarding the potential influences of these factors will be drawn from an assessment of the consistency of the findings of the meta‐regressions that were able to be conducted across the outcomes.

In addition, for each outcome, an assessment of whether there is any evidence of publication bias will be conducted by using the funnel‐plot technique to visually display the distribution of un‐weighted contrast effect sizes by sample size. We will apply Egger's regression asymmetry test to funnel plots to test for funnel plot asymmetry (Egger, Smith, Schneider, & Minder, 1997).

#### Assessment of core programme components (Objective 2)

For all the programme components, to be identified through the analysis of programme descriptions, dummy variables will be created to indicate the presence or absence of each. For each outcome, where there are sufficient studies, a meta‐regression will be run with all of these dummy variables included. As before, and in each case, meta‐regression will only be used if there are the minimum prerequisite of ten studies (Borenstein et al., 2009). The statistical significance of the estimated coefficient for each dummy variable will be used to test formally whether the associated component can be considered to be an ‘active ingredient’ or not. Standardised betas will then be used to assess the relative importance of each of these ‘active ingredients’.

#### Assessment of potential influence of programme fidelity (Objective 3)

For each outcome, where there are sufficient studies, a meta regression will be run that will include the measure of programme fidelity added as an independent variable. The statistical significance of the estimated coefficient(s) for this independent variable will be used to test formally whether programme fidelity has an influence on the effectiveness of SEL programmes. If the estimated coefficient is found to be statistically significant, it will then be used to illustrate the size of the influence of programme fidelity.

#### Assessment of differential effectiveness in relation to age, gender and socio‐economic background (Objective 4)

Eligible studies will be coded in terms of:

- whether the age of the participants falls into preschool (typically aged 3–5), lower elementary/primary school (typically aged 5–8) or upper elementary/primary school (typically aged 8–11).
- the effects for boys and girls where reported separately. In such cases, each study will generate two studies for the purposes of the subgroup analyses to follow (one based upon the sample for boys only, and the other for girls only).
- whether the participants are largely from economically deprived/disadvantaged neighbourhoods or not.

Three subgroup analyses will then be conducted in relation to each of the three factors above (age, gender and socio‐economic background) for each of the primary and secondary outcomes. The subgroup analyses (based upon random‐effects models), will group studies by sub‐category and estimate overall effects sizes for each.

### Treatment of qualitative research

This systematic review is limited to synthesising the available evidence on the effectiveness and of universal school‐based social and emotional learning programmes. It is beyond the remit of this present review to attempt to also synthesise the associated evidence related to process evaluations of such programmes. As such, there is no plan to include qualitative research.

## REVIEW AUTHORS

**Lead review author:** *The lead author is the person who develops and co‐ordinates the review team, discusses and assigns roles for individual members of the review team, liaises with the editorial base and takes responsibility for the on‐going updates of the review.*

**Table:** 

**Name:**	**Paul Connolly**
Title:	Professor
Affiliation:	Dean of Research, Faculty of Arts, Humanities and Social Sciences Director, Campbell Centre UK & Ireland Professor, School of Social Sciences Education and Social Work.
Address:	Queen's University Belfast, University Road
City:	Belfast
Postal Code:	BT7 1NN
Country:	Northern Ireland
Phone:	+44 (0)28 9097 3991
Email:	paul.connolly@qub.ac.uk
Co‐author:	
Name:	**Sarah Miller**
Title:	Dr
Affiliation:	Deputy director, Campbell Centre UK & Ireland Centre for Evidence and Social Innovation Senior lecturer, School of Social Sciences Education and Social Work.
Address:	Queen's University Belfast, University Road
City:	Belfast
Postal Code:	BT7 1NN
Country:	Northern Ireland
Phone:	+44 (0)28 9097 5944
Email:	
Co‐author:	
Name:	**Jennifer Hanratty**
Title:	Dr
Affiliation:	Campbell Centre UK & Ireland Centre for Evidence and Social Innovation School of Social Sciences Education and Social Work.
Address:	Queen's University Belfast, University Road
City:	Belfast
Postal Code:	BT7 1NN
Country:	Northern Ireland
Phone:	028 9097 2593
Email:	j.hanratty@qub.ac.uk
Co‐author:	
Name:	**Jennifer Mooney**
Title:	Dr
Affiliation:	Lecturer, Queen's University Belfast, University Road
Address:	Belfast
City:	BT7 1NN
Postal Code:	Northern Ireland
Country:	Queen's University Belfast, University Road
Phone:	02890973177
Email:	j.mooney@qub.ac.uk
Co‐author:	
Name:	**Seaneen Sloan**
Title:	Dr
Affiliation:	Lecturer, University College Dublin
Address:	School Of Education, Roebuck Offices, Belfield
City:	Dublin
Postal Code:	Dublin 4
Country:	Ireland
Phone:	+353 1 7167987
Email:	Seaneen.sloan@ucd.ie

## ADVISORY GROUP

Members of the current Review Team (Connolly, Miller and Sloan) have been part of a team that has undertaken a large cluster‐randomised controlled trial of the Roots of Empathy social and emotional learning programme in Northern Ireland. This trial has been funded by the National Institute for Health Research (ISRCTN07540423). A Roots of Empathy Regional Planning Group has been established to oversee the delivery of the programme. The Group is led by the Public Health Agency and includes representatives from each of the five Health and Social Care Trusts in Northern Ireland charged with the delivery of the programme.

To supplement the findings of the trial, and to inform future policy decisions regarding the promotion of social and emotional learning in schools, the Public Health Agency has commissioned Connolly and Miller to undertake this present systematic review. The Regional Planning Group has agreed to act as an Advisory Group for the review. They have considered and provided feedback on this protocol. The one change to this review that has been made as a result of this feedback has been to add a further objective to include an appraisal and synthesis of the evidence on the cost‐effectiveness of social and emotional learning programmes.

The subsequent role of the Advisory Group will be to help interpret the findings of the systematic review and proposed meta‐analysis, particularly in relation to identifying the implications for policy and practice.

## ROLES AND RESPONSIBILITIES

- Content: Connolly, Hanratty, Miller, Roberts, Sloan,.
- Systematic review methods: Hanratty, Miller.
- Statistical analysis: Connolly, Hanratty, Miller.
- Information retrieval: Hanratty, Sloan.

## FUNDING

Review authors Connolly and Miller were awarded funding from the Public Health Agency (Northern Ireland) to conduct this review.

## POTENTIAL CONFLICTS OF INTEREST

None of the review authors has a financial interest in this review. None of them has been involved in the development of interventions to improve social and emotional learning. Some of the authors (Connolly, Miller, Hanratty, Sloan) have either completed and/or are currently running trials of interventions that may fall within the scope of this present review. Connolly is currently supervising a PhD student whose doctoral research involves the development and pilot evaluation of a preschool social and emotional learning programme that may fall within the scope of this present review. Hanratty is currently conducting a Cochrane systematic review of child‐focused interventions for anger and aggression in young children.

## PRELIMINARY TIMEFRAME

Date you plan to submit a draft review: 30 June 2018.

## PLANS FOR UPDATING THE REVIEW

Review authors will update the review five years after the submission date.

## AUTHOR DECLARATION

### Authors' responsibilities

By completing this form, you accept responsibility for preparing, maintaining and updating the review in accordance with Campbell Collaboration policy. The Campbell Collaboration will provide as much support as possible to assist with the preparation of the review.

A draft review must be submitted to the relevant Coordinating Group within two years of protocol publication. If drafts are not submitted before the agreed deadlines, or if we are unable to contact you for an extended period, the relevant Coordinating Group has the right to de‐register the title or transfer the title to alternative authors. The Coordinating Group also has the right to de‐register or transfer the title if it does not meet the standards of the Coordinating Group and/or the Campbell Collaboration.

You accept responsibility for maintaining the review in light of new evidence, comments and criticisms, and other developments, and updating the review at least once every five years, or, if requested, transferring responsibility for maintaining the review to others as agreed with the Coordinating Group.

### Publication in the Campbell Library

The support of the Coordinating Group in preparing your review is conditional upon your agreement to publish the protocol, finished review, and subsequent updates in the Campbell Library. The Campbell Collaboration places no restrictions on publication of the findings of a Campbell systematic review in a more abbreviated form as a journal article either before or after the publication of the monograph version in *Campbell Systematic Reviews*. Some journals, however, have restrictions that preclude publication of findings that have been, or will be, reported elsewhere and authors considering publication in such a journal should be aware of possible conflict with publication of the monograph version in *Campbell Systematic Reviews*. Publication in a journal after publication or in press status in *Campbell Systematic Reviews* should acknowledge the Campbell version and include a citation to it. Note that systematic reviews published in *Campbell Systematic Reviews* and co‐registered with the Cochrane Collaboration may have additional requirements or restrictions for co‐publication. Review authors accept responsibility for meeting any co‐publication requirements.

**I understand the commitment required to undertake a Campbell review, and agree to publish in the Campbell Library. Signed on behalf of the authors**:

**Table:** 

**Form completed by:** Paul Connolly	**Date:** 5 January 2018

## Appendix

Searched in all text fields unless otherwise specified.

### Participants

In order to identify studies that concern children in a school setting we combine broad terms for age with (school* or class or classroom). This is to improve the precision of the search and avoid returning too many irrelevant records concerning older children, or papers concerning other schools settings, for example medical school, law school. The search strings for participants therefore come in two parts;

(Child* or youth* or boy* or girl* or Young children or Young people or preadolescen* or pre‐adolescen* or early adolescen* or earlyadolescen* or Junior infant* or senior infant* or “reception class” or First Class or Second Class or Third Class or Fourth Class or Fifth Class or Sixth Class or Grade* 1 or Grade* 2 or Grade* 3 or Grade* 4 or Grade* 5 or Grade* 6 or Intermediate grades or Grade* one or Grade* two or Grade* three or Grade* four or Grade* five or Grade* six or First Grade or Second Grade or Third Grade or Fourth Grade or Fifth Grade or Sixth Grade or 1st grade or 2nd grade or 3rd Grade or 4th Grade or 5th grade or 6th Grade or Age* 3 or 3 year* old* or Age* 4 or 4 year* old* or Age* 5 or 5 year* old* or Age* 6 or 6 year* old* or Age* 7 or 7 year* old* or Age* 8 or 8 year* old* or Age* 9 or 9 year* old* or Age* 10 or 10 year* old* or Age* 11 or 11 year* old*) AND school* or class or classroom

OR

prekindergar* or pre‐kindergar* or kindergar* or pre‐k or pre‐school* or preschool* or early childhood education* or (Nursery* adj1 (class* or school* or student* or pupil* or education)) or (elementary adj1 (class* or school* or student* or pupil* or education)) or (primary adj1 (class* or school* or student* or pupil* or education))

### Study design

(singl* or doubl* or trebl* or tripl*) adj3 (blind* or mask*)) OR

Allocation or allocated or assigned or Causal Design or Cluster Design or Comparison Condition or Comparison group or Control condition or Control Group or Control groups or Controlled trial or cluster trial or random* or RCT or clinical trial OR

((evaluat* or prospective*) adj3 (study or studies)) OR

((Process Evaluation or Program* evaluation)) OR

((Treatment Condition or Treatment effectiveness evaluation or Treatment group or Treatment outcomes))

### Social‐emotional terms

Terms presented alphabetically on separate lines for clarity, all terms combined with “OR”

(Acting out)

(adjustment adj1 (disorder* or emotion* or school))

(aggression or aggressive behavio* or anger)

(attachment adj1 (problem* or disorder*))

(behavio* adj1 (anti‐social or antisocial or development or difficult* or management or problem* or high risk or risky or social or prosocial or pro‐social or helping))

(bully*)

(cognitive function or conflict management or conflict resolution or coping or delinquen*)

(communicat* adj1 (skill* or problem* or interpersonal))

(emotion* adj1 (control or regulat*))

(emotional intelligence or emotional wellbeing or managing feelings)

(empathy or executive function or hostility or impulsiv*)

(interpersonal adj1 (competence or conflict or interaction or relation* or skill*))

(mental health)

(mindfulness)

(non‐cognitive skill)

(peer adj1 (relation* or acceptance or rejection or pressure))

(perspective taking or positive youth development or pro?social or problem solving or relationship skill*)

(resilience or resiliency) or grit or character development or 21st century skill

(self adj1 (control or inhibition or awareness or efficacy or esteem or perception or regulat* or respect))

(social adj1 (adjust* or alienat* or emotion* or attitud* or cognition or competenc* or development or interaction or isolation or learn* or participation or problem* or skill*))

(social and emotional learning) or socio‐emotional learning

(stress adj1 (manag* or reduc* or control))

(violence or violent)

### Additional limits

Due to the very large number of records (>13,000) returned when testing the searches in PsycInfo, additional limits were added by combining the strings above AND the following;

“Curriculum & Programs & Teaching Methods”.cc or (intervention* or program* or course* or polic* or practice* or curricul* or environment* or prevent* or training or treat* or school‐based or school based or class based or classroom based or class‐based or classroom‐based or universal)

### Known interventions

Finally, we added a search for the following known interventions;

“Roots of Empathy” OR PATHS OR “promoting alternative thinking strategies” OR “Skills for Life” OR “The Good Behavio* Game” OR “Bounce Back” OR “Zippys Friends” OR “RTime” OR “Circle Time” OR “Primary SEAL” OR “Positive Action” OR “Incredible Years” OR “Tools of mind” OR “Head Start REDI” or “incredible years”.

For all strings proximity searching will be employed using notation appropriate for each database (e.g. “primary adj1 school”, “fourth N1 grade” to limit the number of irrelevant records retrieved.

## 1.1 Trial/Study Information

**Table:**

Enter new trial ID	**Radio**
Author contact details Preferably email address, google to find authors if not reported in the paper.	**Text**
What year was the report produced?	**Text**
Original Language	**Radio**
Source of the report (according to the reference)	**Text**
Source of the report	**Radio**
Does the trial have a clinical trial reference and/or protocol information?	**Radio**
Who funded the trial? If the evaluation and the intervention were funded by different sources please extract details as reported	**Text**
Is the funder independent of the evaluators? Look at who wrote the report and any affiliations to the funder or any declarations of interest in the document, on the funders website, authors website etc. Provide information or quote from the report to justify the assessment of funder and evaluator independence	**Radio**
Developer involvement Base your assessment on the authorship of the paper/report, declaration of conflict of interests and the methods section. Provide the information on which you based your decision.	**Radio**
Is this an efficacy (ideal conditions) or effectiveness (real world) trial? Select the description that most closely matches the trial as described Efficacy: The trial schools or teachers received support, training, staff or resources that would not normally be available to the organisation purchasing/adopting the intervention Effectiveness: The intervention was conducted in naturalistic settings without developer/researcher support, using just the staff and resources that would be normally available	**Radio**
Who was consent sought from? Check all the apply	
District/local authority	**Checkbox**
School principal/board	**Checkbox**
Teacher	**Checkbox**
Parent	**Checkbox**
Child	**Checkbox**
When was consent to participate obtained?	**Radio**
Was consent opt in or opt out?	**Radio**
What year did the trial begin and end?	**Text**
Was this a cluster randomised or randomised controlled trial? Any other study design is not eligible for this review. Designs described as quasi‐randomised can be included subject to an assessment of the randomisation procedure used	**Radio**
Unit of Randomisation	
Pupil	**Checkbox**
Teacher	**Checkbox**
Classroom	**Checkbox**
Year Group	**Checkbox**
School	**Checkbox**
School cluster/federation	**Checkbox**
District/local authority	**Checkbox**
Other	**Checkbox**
How many arms (groups) in the trial? All trials need to have at least two arms, intervention and control. Add additional groups as necessary	**Radio**
Group 1 Select the group type (intervention or control group) and then label the group	**Radio**
Group 2 Select the group type (intervention or control group) and then label the group	**Radio**
Intervention	**Checkbox**
Control	**Checkbox**
In which country or countries did the trial take place?	**Checkbox**
Number of sites	**Text**
What kind of locations was the intervention delivered in?	**Radio**
Did the trial take place in the country where the programme/intervention was developed? Home: Conducted in the same country Transported: Conducted in a different country without any substantive modifications (other than translation into local language) Away: Conducted in a different country and adapted to suit the local culture	**Radio**
Which types of schools recruited for the trial?	
pre‐school (pre‐k, nursery etc., typically age 3)	**Checkbox**
early primary (KS 0, typically age 4–6 years)	**Checkbox**
mid primary (KS 1, typically 6–8 years)	**Checkbox**
late primary (KS 2, typically 8–11 years)	**Checkbox**
Did the trialists target particular schools or areas, for example, areas of high deprivation or greater numbers of BME students?	**Text**
How were participants/schools recruited? for example via local authorities, flyers and posters, national media etc.	**Text**
Extract details of all inclusion/exclusion criteria Include cluster and individual level criteria Interventions can still be universal if they include children who represent the general population. Excluding children who have a particular diagnosis or disorder is acceptable but should be noted.	**Text**
Was participation voluntary? Assess whether schools/individuals were obliged to participate or given some incentive or participated voluntarily	**Radio**
How were children chosen to participate? Extract any text describing how children were chosen and note the page number quoted from	**Radio**
Ineligible participants were identified… Before recruitment (not asked to participate) After recruitment before randomisation (not assigned to a group) After randomisation (assigned to a group but did not receive the intervention) After intervention delivered (assigned to a group, received the intervention or control condition but excluded from analysis)	**Radio**
Is there any suggestion in the document or in the data that the distribution of exclusions differed between groups? For example, more children excluded from the intervention group because they exhibited extreme behaviour	**Radio**
Were participating schools/children matched?	**Radio**
At what level were participants matched? Select all that apply	
District/area/local authority	**Checkbox**
School	**Checkbox**
Teacher	**Checkbox**
Classroom	**Checkbox**
Pupil	**Checkbox**
What variables were used to match participants?	
Age	**Checkbox**
Gender	**Checkbox**
% Free school meals	**Checkbox**
Area level deprivation	**Checkbox**
Geographic location	**Checkbox**
Size of school	**Checkbox**
Ethnicity	**Checkbox**
% students with English as second language	**Checkbox**
not reported	**Checkbox**
School information size, demographics, %FSM, etc.	**Text**
Clinical characteristics of group (diagnosis, CD, ODD etc.) This is only relevant for the ANGER REVIEW	**Text**
Class/grade level as reported	**Text**
Are there some children included in the trial who are under 3 or over 11? You can tell by looking at the age range if reported of check the average age and standard deviation to infer the age range (e.g. a mean of 11 and SD of 4 indicates that many will be over our age limit)	**Text**
Race & Ethnicity Extract any description of the race/ethnicity of the sample	**Text**
Socio‐economic status Extract any detail on the samples socioeconomic status, e.g. parental occupational class, % receiving Free school meals	**Text**
Parent information Extract any detail given on parents e.g. single parent families, parent education If not described use “not reported”	**Text**
Extract any further detail E.g. age at trial entry or age at follow‐up? Is outcome data broken down by age group or age analysed as a mediator of intervention effect?	**Text**
Does the report include a flow chart illustrating number of participants at each stage (e.g. recruited, randomised, received the intervention, completed outcome measures)?	**Radio**
Participant numbers (extracted for overall sample and each group where reported)	
Screened	**Checkbox**
Eligible	**Checkbox**
Randomised	**Checkbox**
Baseline	**Checkbox**
Post‐test	**Checkbox**
First follow‐up	**Checkbox**
Final follow‐up	**Checkbox**
Age (extracted for overall sample and each group where reported)	
Mean Age	**Checkbox**
Standard Deviation	**Checkbox**
Range	**Checkbox**
Gender (extracted for overall sample and each group where reported)	
Number female	**Checkbox**
Number male	**Checkbox**
% Female	**Checkbox**
% Male	**Checkbox**
Race (extracted for overall sample and each group where reported) Indicate whether the sample comprises 80% or more of the ethnic majority group of the country this is usually white/Caucasian for American/European studies 80% or more ethnic minority group members If no group comprises 80% or more of the sample, mark as Mixed.	**Radio**
SES Status (extracted for overall sample and each group where reported) Indicate whether the sample is described as comprising 80% or more; Low SES (at or below poverty line or receiving FSM) Working class or lower middle class Middle or upper class If no group comprises 80% or more of the sample, mark as Mixed.	**Radio**
Drop out/attrition Extract all information on people who dropped out, at what stage they dropped out and why. If no information is given write “not reported”. If there are no drop outs at any stage, everyone who began the trial was still included in the final follow‐up and analysis then write “no apparent drop outs”	**Text**

## 1.2 Intervention Information

**Table:**

Do you want to extract data for an intervention or control/comparison group?	**Radio**
Name of the Intervention	**Text**
Description of the intervention Describe the intervention and each component as described by the authors	**Text**
What is the primary aim of the intervention as stated by the authors?	**Text**
What is the secondary aim of the intervention? If none is given by the authors write “none given”	**Text**
What is the theory of change? Extract the author's description of how the intervention is supposed to improve things for children	**Text**
Identify all major components of the intervention Add new components to the list where necessary	**Checkbox**
Communication skills training	
What specific risk factors or skills does the intervention target? Add to the list when necessary	**Checkbox**
Social competence	
Is there any evidence that the intervention is developmentally appropriate? For example, the authors make reference to developmental or developmental stages of the target group.	**Checkbox**
Yes	
No	**Checkbox**
Not clear	**Checkbox**
Target of intervention N.B. all must include a pupil level element	
Standalone curriculum for pupils	**Checkbox**
SEL integrated into the curriculum	**Checkbox**
Classroom level, non‐curricular change e.g. class behaviour rules	**Checkbox**
Teacher training/behaviour change	**Checkbox**
School level reinforcement of skills (e.g. school assemblies, playground rules)	**Checkbox**
School system/structural change (school policies, school day reform)	**Checkbox**
Parent involvement	**Checkbox**
School‐community involvement	**Checkbox**
Individual Training	**Checkbox**
School Setting	**Checkbox**
In classroom (whole class)	
In classroom (pull out group)	**Checkbox**
Outside classroom (whole class)	**Checkbox**
Outside classroom (pull out group)	**Checkbox**
School level (e.g. school assemblies etc.)	**Checkbox**
Is the intervention delivered at a group or individual level?	**Checkbox**
Group	
Individual	**Checkbox**
Both	**Checkbox**
Duration/course of the intervention from beginning to end (in weeks) Note. Consider a school semester to be 18 weeks. A school year is 36 weeks	**Text**
Average length of each session (in minutes)	**Text**
Number/frequency of sessions	**Text**
Timing/intensity of the intervention delivery	**Radio**
Were booster sessions delivered some time after the intervention ended?	**Radio**
Total length of the intervention (in hours)	**Text**
Sequenced: Does the program use a sequenced set of activities to achieve their objectives relative to skill development? Note. The presence of a program manual, or set of lesson plans signals Yes for this item. Several reports describe the use of “structured” skill activities. If so, also score Yes. If the report only mentions the name of a program or set of activities with which you are not familiar, write the term down and we will discuss it.	**Radio**
Note. For programs attempting to promote self‐esteem or cultural identity, the “skill” involved is a bit different. Are there any indications or explanations in the report of how the program activities are connected and build on each other to achieve their desired goal? Do youth reflect on their actions or performance, are they asked to consider how it pertains to who they are and what positive features they possess? If the report only speaks generally about activities, e.g., recreational, youth development, field trips, etc., then code No.	
Active: does the program use active forms of learning to help youth learn new skills? What qualifies as active forms of learning? In general, youth must act on the material, try new behaviours, participate in role plays, or do behavioural rehearsal when practising new skills. Hands‐on forms of learning are used. Youth learn by doing. They practice doing new things as opposed to passive forms of learning that emphasise didactic instruction, lectures, or general discussions in which children primarily talk, but do not practice new behaviours. Also look for indications of live or media modelling of the desired behaviours Note. Do not score yes if most activities are lecture‐oriented (didactic) or discussion‐oriented	**Radio**
Focused: does the program have at least one component focused on developing personal or social skills? Does the program devote some time and activities specifically or primarily to promoting personal or social skills?	**Radio**
Explicit: does the program target specific personal or social skills? Can you tell what specific personal or social skill youth are expected to acquire in the program? Look for instances where the personal or social skills in our coding manual are identified. Self‐esteem, self‐concept, racial or cultural identity, interpersonal problem solving, refusal skills, coping strategies, and so on.	**Radio**
Who delivered the intervention? For this code, a researcher may serve (and usually does) as a consultant or trainer, but we want to code who directly delivered the intervention to the children.	**Checkbox**
Classroom teacher	
Trained researcher	**Checkbox**
Multiple school personnel	**Checkbox**
Qualified clinical therapist	**Checkbox**
Trained student therapist	**Checkbox**
Trained lay person	**Checkbox**
Parents	**Checkbox**
Other	**Checkbox**
Who trained the person/people delivering the intervention?	**Text**
Training/qualification of the person/people delivering the intervention?	**Text**
Is the intervention manualised?	**Text**
Was there any assessment of fidelity? IF YES GIVE DETAILS Fidelity refers to the intervention being delivered the way it was intended. Fidelity checks may include checking that all lessons were delivered, that there was no deviation from the manual, there was regular supervision of staff to make sure things were running as intended.	**Radio**
Were any issues with fidelity or implementation reported?	**Checkbox**
Yes	
No	**Checkbox**
Not reported	**Checkbox**
Detail any supervision of intervention staff? Who supervised and what did the supervision consist of?	**Text**
Were any concurrent interventions being delivered? Either to both intervention and control or to one group only? E.g. schools entering into collaboration, schools establishing a counselling service at the same time. Anything mentioned that could impact on the outcomes of interest	**Text**
What kind of control group is this? Is it a waitlist control, treatment as usual, comparison to another active intervention, comparison between irrelevant intervention and irrelevant intervention plus a relevant one (e.g. roots of empathy plus parent training vs parent training only?)	**Checkbox**
Waitlist	
Treatment as usual	**Checkbox**
Another inactive intervention	**Checkbox**
Another active intervention	**Checkbox**
No treatment concurrent control	**Checkbox**
Irrelevant intervention	**Checkbox**
Description of what happens to the control group Describe what happens to the control group in the study Often little information is given but extract any info – it will be useful later for deciding what interventions/studies are comparable and can and cannot be combined in meta‐analysis.	**Text**

## 1.3 Cochrane Risk of Bias Tool

**Table:**

The investigators describe a random component in the **sequence generation** process such as: Referring to a random number table Using a computer random number generator Coin tossing Shuffling cards or envelopes Throwing dice Drawing of lots Minimization (Minimization may be implemented without a random element, and this is considered to be equivalent to being random.)	**Radio**
The investigators describe a non‐random component in the sequence generation process, for example: Sequence generated by odd or even date of birth Sequence generated by some rule based on date (or day) of admission Sequence generated by some rule based on hospital or clinic record number Or other non‐random approaches for example: Allocation by judgment of the clinician Allocation by preference of the participant Allocation based on the results of a laboratory test or a series of tests Allocation by availability of the intervention	**Radio**
Text fragment from manuscript to support above decision	**Text**
**Risk of Bias in Sequence Generation is:**	**Radio**
Participants and investigators enrolling participants could not foresee assignment because one of the following, or an equivalent method, was used to conceal allocation: Central allocation (including telephone, web‐based, and pharmacy‐controlled, randomization) Sequentially numbered drug containers of identical appearance Sequentially numbered, opaque, sealed envelopes.	**Radio**
Participants or investigators enrolling participants could possibly foresee assignments and thus introduce selection bias, such as allocation based on: Using an open random allocation schedule (e.g. a list of random numbers) Assignment envelopes used without appropriate safeguards (e.g. if envelopes were unsealed or non‐opaque or not sequentially numbered) Alternation or rotation Date of birth Case record number Any other explicitly unconcealed procedure.	**Radio**
Text fragment from manuscript to support above decision	**Text**
**Risk of Bias in Allocation Concealment is:**	**Radio**
Any one of the following is considered adequate blinding: No blinding, but the review authors judge that the outcome and the outcome measurement are not likely to be influenced by lack of blinding. Blinding of participants and key study personnel ensured, and it is unlikely that the blinding could have been broken. Either participants or some key study personnel were not blinded, but outcome assessment was blinded and the non‐blinding of others is unlikely to introduce bias.	**Radio**
Any one of the following is considered inadequate blinding: No blinding or incomplete blinding, and the outcome or outcome measurement is likely to be influenced by lack of blinding. Blinding of key study participants and personnel attempted, but likely that the blinding could have been broken. Either participants or some key study personnel were not blinded, and the non‐blinding of others likely to introduce bias.	**Radio**
Text fragment from manuscript to support above decision	**Text**
**Risk of Bias in blinding of participants, personnel and outcomes assessors is:**	**Radio**
Any one of the following is considered adequate blinding: No blinding, but the review authors judge that the outcome and the outcome measurement are not likely to be influenced by lack of blinding. Blinding of participants and key study personnel ensured, and it is unlikely that the blinding could have been broken. Either participants or some key study personnel were not blinded, but outcome assessment was blinded and the non‐blinding of others is unlikely to introduce bias.	**Radio**
Any one of the following is considered inadequate blinding: No blinding or incomplete blinding, and the outcome or outcome measurement is likely to be influenced by lack of blinding. Blinding of key study participants and personnel attempted, but likely that the blinding could have been broken. Either participants or some key study personnel were not blinded, and the non‐blinding of others likely to introduce bias.	**Radio**
Text fragment from manuscript to support above decision	**Text**
**Risk of Bias in blinding of participants, personnel and outcomes assessors for objective outcomes is:**	**Radio**
Any one of the following is considered adequate blinding: No blinding, but the review authors judge that the outcome and the outcome measurement are not likely to be influenced by lack of blinding. Blinding of participants and key study personnel ensured, and it is unlikely that the blinding could have been broken. Either participants or some key study personnel were not blinded, but outcome assessment was blinded and the non‐blinding of others is unlikely to introduce bias.	**Radio**
Any one of the following is considered inadequate blinding: No blinding or incomplete blinding, and the outcome or outcome measurement is likely to be influenced by lack of blinding. Blinding of key study participants and personnel attempted, but likely that the blinding could have been broken. Either participants or some key study personnel were not blinded, and the non‐blinding of others likely to introduce bias.	**Radio**
Text fragment from manuscript to support above decision	**Text**
**Risk of Bias in blinding of participants, personnel and outcomes assessors for subjective outcomes is**	**Radio**
Any one of the following is considered adequate blinding: No blinding, but the review authors judge that the harms and the harms measurement are not likely to be influenced by lack of blinding. Blinding of participants and key study personnel ensured, and unlikely that the blinding could have been broken. Either participants or some key study personnel were not blinded, but harms assessment was blinded and the non‐blinding of others is unlikely to have introduced bias.	**Radio**
Any one of the following is considered inadequate blinding: No blinding or incomplete blinding, and the harms or harms measurement is likely to be influenced by lack of blinding. Blinding of key study participants and personnel attempted, but likely that the blinding could have been broken. Either participants or some key study personnel were not blinded, and the non‐blinding of others likely to introduce bias.	**Radio**
Provide text fragments from the manuscript or other evidence to support your decision:	**Text**
**Risk of Bias in blinding of participants, personnel and outcomes assessors for HARMS is:**	**Radio**
Were attrition and exclusions reported?	**Radio**
Number of participants excluded from analysis or lost through attrition (or n/a)	**Text**
No missing outcome data Reasons for missing outcome data unlikely to be related to true outcome (for survival data, censoring unlikely to be introducing bias) because; Missing outcome data balanced in numbers across intervention groups, with similar reasons for missing data across groups; For dichotomous outcome data, the proportion of missing outcomes compared with observed event risk not enough to have a clinically relevant impact on the intervention effect estimate; For continuous outcome data, plausible effect size (difference in means or standardized difference in means) among missing outcomes not enough to have a clinically relevant impact on observed effect size; Missing data have been imputed using appropriate methods.	**Radio**
Missing outcome data may introduce bias because; Reason for missing outcome data likely to be related to true outcome, with either imbalance in numbers or reasons for missing data across intervention groups For dichotomous outcome data, the proportion of missing outcomes compared with observed event risk is enough to induce clinically relevant bias in intervention effect estimate For continuous outcome data, plausible effect size (difference in means or standardized difference in means) among missing outcomes enough to induce clinically relevant bias in observed effect size; ‘As‐treated’ analysis done with substantial departure of the intervention received from that assigned at randomization; Potentially inappropriate application of simple imputation.	**Radio**
**Risk of bias from missing outcome data**	**Radio**
Text fragment from manuscript to support above decision	**Text**
The study protocol is available and all of the study's pre‐specified (primary and secondary) outcomes that are of interest in the review have been reported in the pre‐specified way. The study protocol is not available but it is clear that the published reports include all expected outcomes, including those that were pre‐specified (Note to rater: convincing text of this nature may be uncommon).	**Radio**
Not all of the study's pre‐specified primary outcomes have been reported. One or more primary outcomes are reported using measurements, analysis methods or subsets of the data (e.g. subscales) that were not pre‐specified. One or more reported primary outcomes were not pre‐specified (unless clear justification for their reporting is provided, such as an unexpected adverse effect). One or more outcomes of interest in the review are reported incompletely so that they cannot be entered in a meta‐analysis. The study report fails to include results for a key outcome that would be expected to have been reported for such a study.	**Radio**
Provide text fragments from the manuscript or other evidence to support your decision:	**Text**
**Risk of Bias from Selective Outcome Reporting**	**Radio**
The study appears to be free of other sources of bias.	**Radio**
There is at least one important risk of bias.	**Radio**
There is at least one important risk of bias. Select any apparent issues with the study:	
Had a potential source of bias related to the specific study design used; or	**Checkbox**
Stopped early due to some data‐dependent process (including a formal‐stopping rule); or	**Checkbox**
Had extreme baseline imbalance; or	**Checkbox**
Has been claimed to have been fraudulent; or	**Checkbox**
There may be a risk of bias, but there is either:	**Radio**
Provide text fragments from the manuscript or other evidence to support your decision:	**Text**
